# Supplementary material for: Thermal laser ablation with tunable lesion size reveals multiple origins of seizure-like convulsions in Caenorhabditis elegans
Source: Sci Rep. 2021 Mar 3;11:5084. doi: 10.1038/s41598-021-84516-y (PMC7930185; doi:10.1038/s41598-021-84516-y)
Supplement: Supplementary file 3 — Supplementary Information 3. [file 41598_2021_84516_MOESM3_ESM.pdf]

Thermal laser ablation with tunable lesion size reveals multiple origins of seizure-like convulsions in *Caenorhabditis elegans*

Anthony D. Fouad[1], Alice Liu[1], Angelica Du[1], Priya D. Bhirgoo[1], and Christopher Fang-Yen[1,2*]

Supporting Information

Video S1: Convulsions in a YX200 *acr-2(gf)* worm with no lesions. Video acquired using RFP fluorescence optics and shown at 1.5 times the original frame rate. Field of view is approximately 1 mm x 1.5 mm. Whole body convulsion indicated with 'W'.

Video S2: **A YX200 worm with mid-body lesions to both the VNC and DNC exhibiting independent anterior and posterior convulsions**. Anterior of the worm is to the upper left. Video acquired using RFP fluorescence optics and shown at 1.5 times the original frame rate. Field of view is approximately 1 mm x 1.5 mm. Annotations denote convulsions scored as occurring in the anterior ('A') or posterior ('P').
